# Protocol for the development and validation of a risk score to predict risk of adverse events associated with systemic anti-cancer treatment in late-stage lung cancer: Lung Cancer Improved Decisions (LUCID)

**DOI:** 10.1186/s41512-026-00225-y

**Published:** 2026-04-08

**Authors:** Ofran Almossawi, Luke Steventon, Ruth Keogh, Karla Diaz-Ordaz, Zhe Wang, Andrew Challenger, David Dodwell, Martin Forster, Kenneth K. C. Man, Li Wei, Sebastian Masento, Adam Januszewski, Pinkie Chambers

**Affiliations:** 1 Great Ormond Street Hospital, London, UK; 2 UCL Institute of Child Health, London, UK; 3 Research Department of Practice and Policy, UCL School of Pharmacy, London, UK; 4 Medical Statistics Department (Faculty of Epidemiology and Population Health), London School of Hygeine & Tropical Medicine, London, UK; 5 Department of Statistical Science, UCL, London, UK; 6 Nuffield Department of Population Health, University of Oxford, Oxford, UK; 7 University College London Hospital NHS Foundation Trust, London, UK; 8 Barts Cancer Centre, St Bartholomew’s Hospital, London, UK

## Abstract

**Background:**

Less than 20% of patients diagnosed with advanced lung cancer will survive beyond five years and half of these will suffer a serious adverse event (SAE) caused by systemic anticancer therapy (SACT) that will result in a hospital attendance. As multiple different SACT treatments are available for patients, a risk score that predicts the likelihood of a SAE following each type of SACT treatment would improve both communication with the patient and shared decision making with all those involved in delivering care for patients. There are currently no risk scores available for use in those with advanced stage lung cancer.

**Aim:**

The overarching aim of this research is to develop and internally validate a risk score that will calculate the individualised risk of SAEs for different SACT treatments for patients with late stage lung cancer.

**Methods:**

Utilising linked cancer registry data (National Cancer Registration and Analysis Service (NCRAS), England) for over 20,000 late stage lung cancer patients, a risk score will be developed using a multivariable logistic regression model to predict the risk of an acute admission within 30 days of SACT administration. Model performance will be summarised using calibration and discrimination. Internal validation will be used to quantify the degree of optimism due to overfitting, using re-sampling bootstrapping. Heterogeneity will be assessed, and the model will be fine-tuned. Fine-tuning and interrogation will be used to evaluate differences in performance between hospitals. The clinical utility will be assessed through calculating the net benefit in preventing SAEs.

**Conclusion:**

A developed risk score (under each treatment strategy) has real potential to support individualised treatment decisions and optimise management of SACT-induced SAEs for patients and reduce hospital attendances.

## Background

The landscape of cancer drugs, referred to as systemic anti-cancer therapy (SACT), has rapidly evolved over the last two decades resulting in an increase in choice of treatments for patients with cancer. Despite these treatment advances, around 10 million patients will die of cancer each year worldwide [1]; this number is projected to increase further, including in lung cancer [2]. In the UK, lung cancer is the second most common cancer in both men and women and has the highest cancer mortality (35,000 patients a year) [3]. Furthermore, by 2060, lung cancers are predicted to be the single greatest contributor to the burden of serious health-related suffering, among cancer patients [4].

Currently, it is anticipated that only 16% of patients diagnosed with lung cancer will survive more than 5 years [5]. This low long-term survival is partly a reflection of the population (i.e. older patients, smoking status) and late-stage at diagnosis [6]: 21% and 50% of patients are diagnosed at stages 3 and 4, respectively, with stages 3b and 4 considered advanced disease and non-curable [3].

In England, 21,652 patients diagnosed with a non-curable lung cancer (i.e., stage IIIb, IIIc and IV) will be started on SACT [7, 8]. The choice of SACT (which currently includes chemotherapy, immunotherapy, targeted therapy or combinations) is guided by the type of lung cancer, stage of disease, evidence from trials, available genomic information and many other individual factors such as age, previous admissions, and other co-morbidities present [9, 10].

Decisions are made by a multidisciplinary team based on the individual patients’ ability to tolerate adverse effects (AE) that are common to the SACT prescribed. The goal of SACT is to improve and retain a patients’ quality of life (in addition to extending survival) and treatments that are likely to result in Serious Adverse Events (SAEs) [11] i.e. requiring a hospital attendance should be avoided. However, around half of patients treated with SACT will still have an AE related hospital admission [12]. Decisions around choice of SACT in patients with advanced lung cancers are time sensitive, complex and need to be made by clinicians with patients based on individualised risks and benefits of treatment [10]. Decisions are becoming more complex as options for different classes of SACT treatment are increasing. Increasing demands of SACT utilisation with an ageing population, increased treatment options coupled with a shortfall in oncologists, nurse specialists and pharmacists [2] means that this research, to reduce iatrogenic hospitalisations, is important to patients, health professionals and policymakers.

Clinical prediction models aim to predict current or future outcomes of patients conditional on a set of covariates that are considered prognostic of the intended outcome. For this purpose, and depending on the type of outcome (binary or continuous), either a logistic or linear regression model is typically fitted, and predictions are made based on these models for a future population. Most clinical prediction models tend to fail when applied to treatment decisions [13], in part due to poor reporting standards [14], but mainly because the models are then applied to populations under different conditions than the ones they were trained on. In the target population, the knowledge from the prediction model is used to inform treatment choice, and this changes the distribution of treatments in the population. These “pure prediction” models were not designed to quantify the outcome that would have occurred had the patient received a different treatment. Instead, they provide a prediction of risk under current or observed conditions, not under hypothetical alternatives, i.e. predicting under the assumption that the relationship between the variables and the outcomes do not change. Therefore the type of patient that opts for a particular treatment or other has different distribution of characteristics, thus not reflecting this “distorts” the predictions. Under current observed conditions, current treatment allocation reflects confounding by indication, and ignoring this in a prediction model yields “spurious” associations reflected in the models.

To overcome this limitation in prognostic research, there has been an increasing focus on counterfactual prediction models. In a counterfactual prediction model, the treatment choice is explicitly incorporated into the model, and the goal is to estimate what the patient's risk would have been under each possible treatment scenario—whether or not that treatment was actually received. This allows the model to answer questions of the form: "What would the outcome have been if this patient had received Treatment A instead of Treatment B?" Such models are grounded in causal inference theory and typically rely on assumptions such as conditional exchangeability (no unobserved confounding), consistency, and positivity. Unlike standard prognostic models, counterfactual models are designed to support individualized treatment decisions by comparing potential outcomes across different treatment strategies, thereby facilitating treatment selection based on predicted benefit. [13].

These counterfactual prediction models need appropriate performance assessment tools. We will implement the performance assessment tools developed by Boyer et al. [14].

There are numerous treatment options available for different subtypes of NSCLC. Tumour histopathological classification and genomic profiling will guide a clinician in determining a treatment algorithm for a patient. For patients with NSCLC without actionable genomic alterations (AGA) there remains a choice of SACT to be made. The choice of SACT depends on evidence of effectiveness of treatment, understanding of patient tolerance and individualised choice. For the case of non-AGA associated NSCLC, treatment choice is single agent SACT (e.g. carboplatin) for those where it is inferred that there will be a poorer tolerance by the patient, two or more cytotoxic agents (e.g. gemcitabine and carboplatin), immune checkpoint inhibitor only (e.g. pembrolizumab) or cytotoxic chemotherapy plus immunotherapy (e.g. carboplatin, pemetrexed and pembrolizumab).

The planned study would improve SACT treatment decision making through better understanding of individualised risk of SAE under different treatments for incurable non-small-cell lung cancer (NSCLC) patients. A counrterfactual risk score may lead to a number of benefits including:

1. Clinicians could more accurately inform patients of the SAE risk that each treatment would carry. Currently general information is given on possible SAEs but not the individualised likelihood or severity, and crucially not from a causal perspective.
2. Multi-disciplinary teams would have a shared understanding of SAE risk; this could aid a more refined shared decision-making approach.
3. Hospitalisation rate due to SAE in this group of patients may be reduced, particularly relevant to minimise patient suffering during their last years of life.
4. A real world application of rigorous counterfactual prediction methods

The remainder of our protocol is based on the HARPER template [15].

## Population and eligibility criteria

Objective: Develop and internally validate a risk score that will calculate the individualised risk of hospitalisations for different SACT treatments for patients with a diagnosis of NSCLC.

Eligibility criteria: We included patients who are 18 years and older, diagnosed with advanced non-AGA associated non-small cell lung cancers between 2016 and 2019 (4-year period).

Exposure and comparator: First exposure to SACT therapy, with four groups of regimens compared against each other.

Outcome: Any unplanned or emergency hospital admission occurring within 30 days of first SACT administration in England expressed in percentage. This is a binary outcome with patients either having an unplanned or emergency admission or not.

Secondary research question and objective: Descriptive Kaplan Meier plots for the outcome of death within 2 years from first dose of SACT (${T}_{0}$) compared between the four treatment algorithm groups. We chose the two-year survival due to the poor prognosis of the population of our study [5].

## Methods

### Study design

#### Rationale for study design choice

**Fig. 1:**
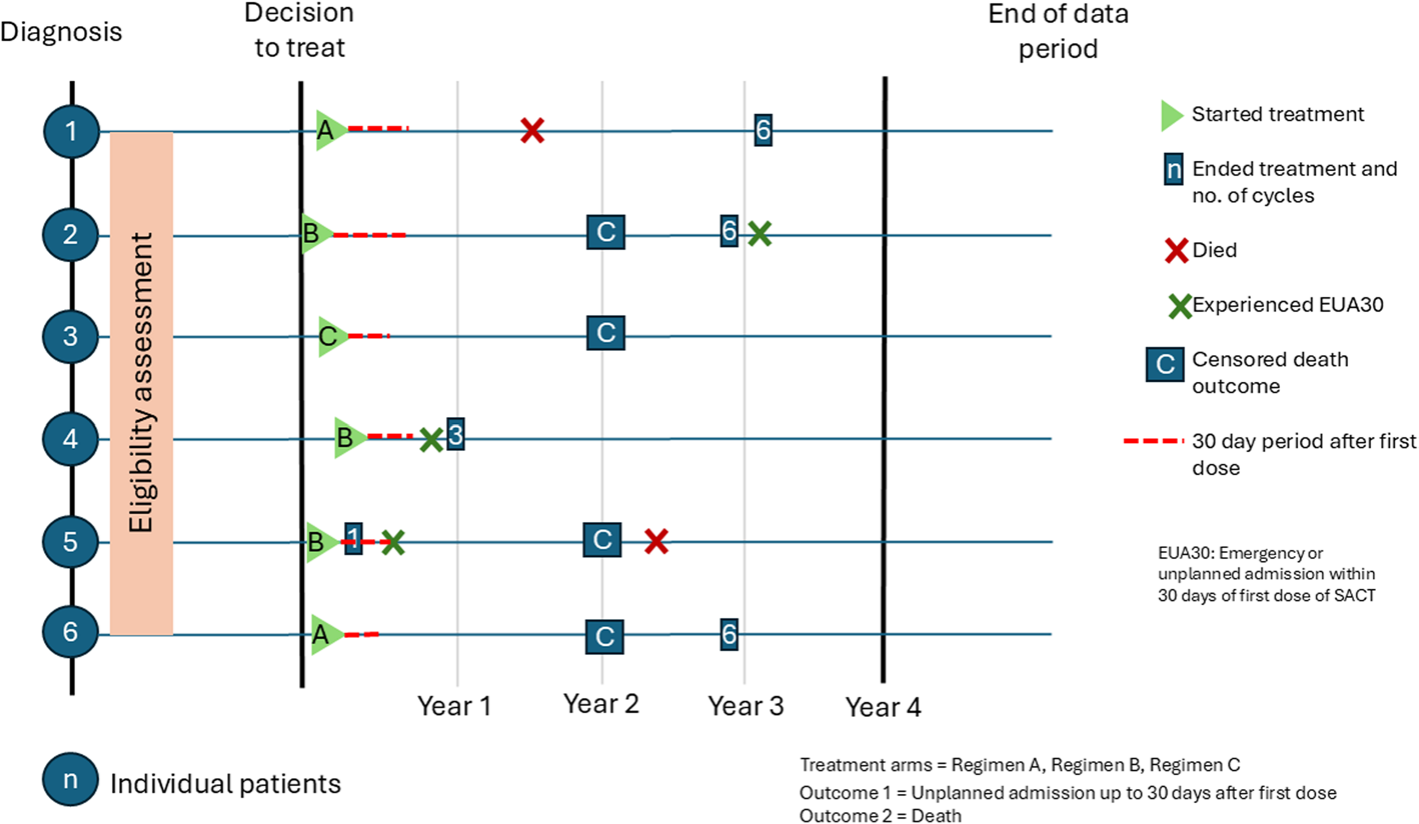
Study design diagram. The numbered circles to the left of the diagram denote individual patients with different courses of treatments and outcomes over the study period. The eligibility criteria for the study participants will be assessed prior to the decision to treat appointment, which is defined as the appointment immediately prior to the first dose of SACT

The American Joint Committee on Cancer (AJCC) recommends the development of outcome prediction models towards achieving personalised treatments for patients. The AJCC published a checklist for outcome prediction models to ensure dependable prediction accuracy in the patient population for which the outcome prediction model was designed [16]. The study design is detailed in Fig. 1.

**Fig. 2:**
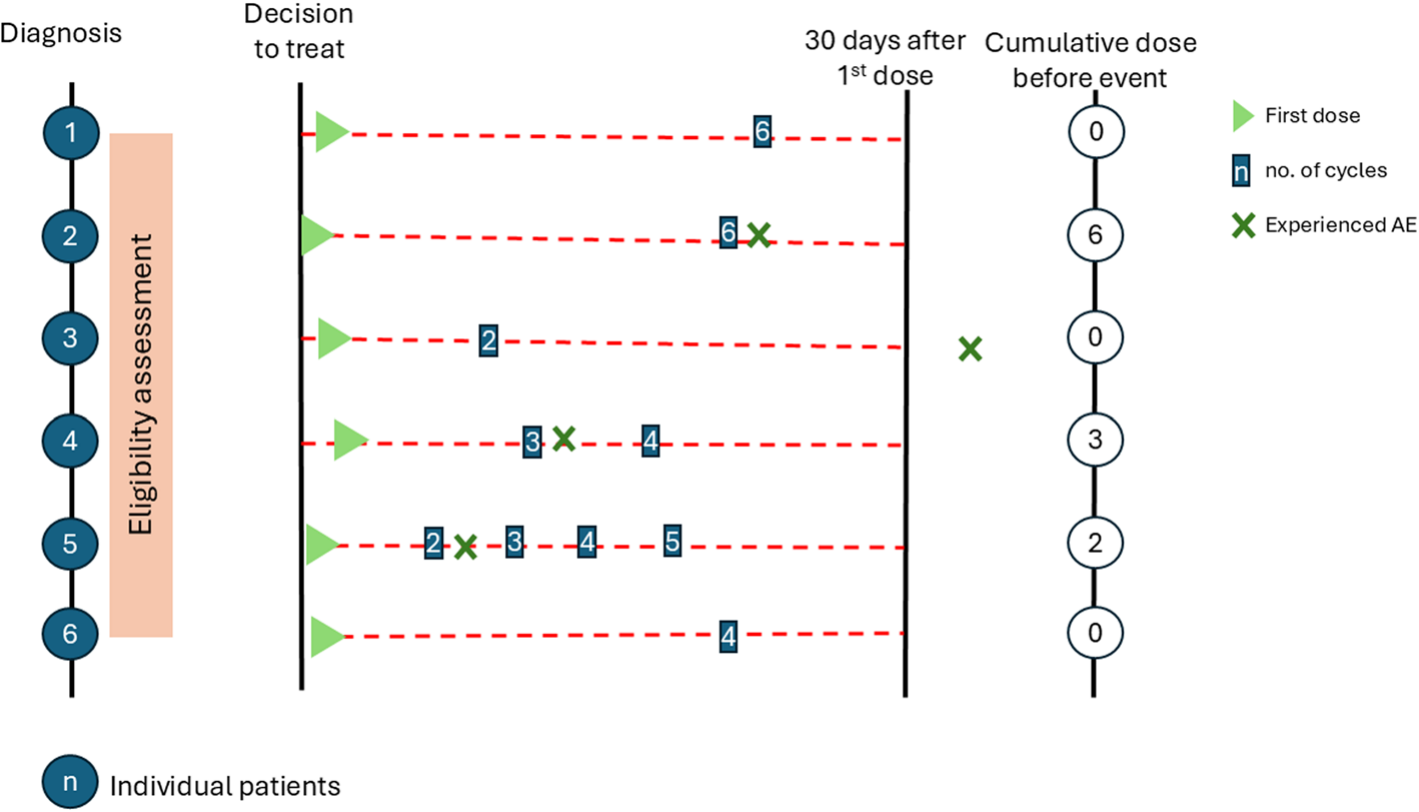
Number of doses received prior to the primary event. For patients 4 and 5, the cumulative dose is three doses and two doses respectively

To determine if the primary event occurred within 30 days of the first dose of SACT, we calculated the number of SACT doses received prior to the first unplanned or emergency admission within the 30 day period after the first dose. Figure 2 below illustrates the cumulative dose for patients prior to the primary event within 30 days of receiving their first dose of SACT. For patients 4 and 5, the cumulative dose is three doses and two doses respectively.

During the eligibility period, patients will have a choice of four treatment protocols (treatment regimens). These are labelled A, B, C, and D, which are defined as:

- Regimen A: Combination chemotherapy alone – inclusive of combination treatments of platinum, gemcitabine, taxanes and any other cytotoxic regimen.
- Regimen B: Combination chemotherapy plus immunotherapy – As regimen A plus the addition of a PD-(L)1 check point inhibitor.
- Regimen C: Immunotherapy only – Any PD-(L)1 checkpoint inhibitor or combination of two.
- Regimen D: Single agent carboplatin.

### Context and rationale for definition of time 0 (and other primary time anchors) for entry to the study population

From the study design diagram, ${T}_{0}$ starts at the date of first SACT dose. Follow up for the primary outcome ends at an event, death or end of study period. An event of EUA30 will mark the end of follow period for the primary research question, ie outcome 1. Patients who develop an EUA30 (outcome 1) will still contribute to the survival analysis (outcome 2, death).

### Context and rationale for study inclusion criteria

The inclusions and exclusions are detailed in Tables 1 and 2 below.

**Table 1:** Operational Definitions of Inclusion Criteria

Criterion	Details	Order of application	Care Settings^1^	Code Type^2^	Diagnosis position	Source for algorithm
Diagnosis	NSCLC	Stage IIIb/c/IV	IP, DC	ICD10	Primary or secondary	SACT database
Number of treatment cycles	At least 1 cycle of treatment	N/A	IP, DC	N/A	N/A	SACT database
Baseline covariates	See DAG in Sect. 7.4 and Appendix 2	All measured at baseline	IP, DC	N/A	N/A	HES APC database and SACT database
Follow up time	30 days for primary outcome (EUA30) 2 years for secondary outcome (death at 2 years)	N/A	IP, DC, ED	Date of unplanned admissions from HES ACP, date of emergency admission from HES AE	N/A	HES APC HES AE

*NSCLC* Non-small cell lung cancer, *SACT* Systemic anticancer therapy, *HES* Hospital episode statistics, *APC* Admitted patient care, *AE* Accident & emergency

^1^*IP* inpatient, *OP* outpatient, *ED* emergency department, *DC* daycare

^2^See appendix for listing of ICD10 codes

**Table 2:** Operational Definitions of Exclusion Criteria

Criterion	Details
Age	< 18 years
Did not receive SACT	Patients with a NSCLC diagnosis and no SACT record
No linkage to HES	Patients identified in NCRAS, with no corresponding record in HES
Not treated within 84 days of diagnosis	If first dose of SACT falls outside of 84 days from diagnosis, this is unlikely to be related to the diagnosis in question

*NSCLC* Non-small cell lung cancer, *NCRAS* National cancer registration and analysis service, *SACT* Systemic anticancer therapy, *HES* Hospital episode statistics

**Fig. 3:**
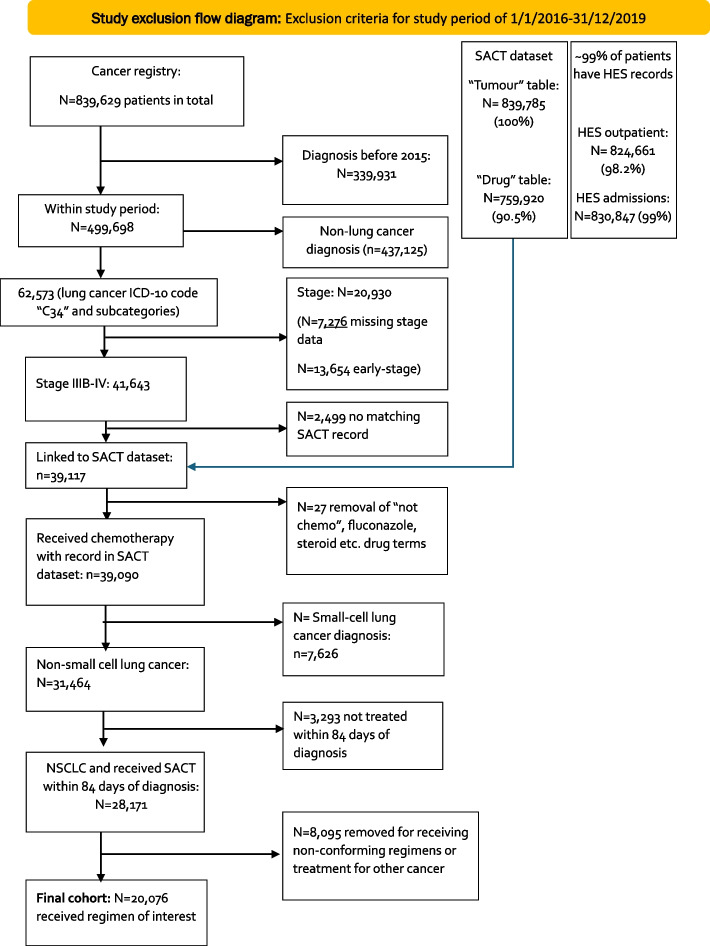
Context and rationale for study exclusion criteria

Figure 3 below shows the data audit that took place prior to the project initiation to support the development of the protocol.

#### Variable selection

**Fig. 4:**
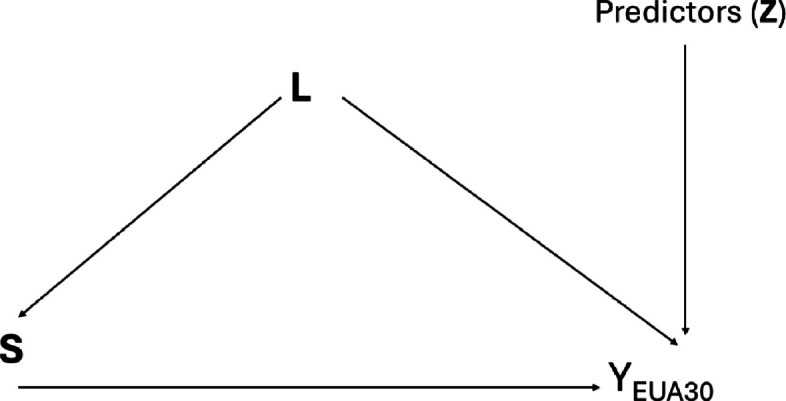
**S** = A, B, C, or D, one of four treatment algorithms as detailed in section Rational for study design choice. YEUA30 is the outcome of adverse event 30 days after administration of chemotherapy. **L** is a vector of variables that are the baseline characteristics considered sufficient to account for confounding. The predictors, **Z**, are the variables that predictive of the outcome but are not related to the treatment.

The variables are grouped into confounders (**L**) and predictors (**Z**). The directed acyclic graph (DAG) in Fig. 4 is a simplified depiction of the relationship between the SACT exposure (**S**), the outcomes (**Y**), and the confounders and predictors. Some of the variables in **L** will also be genuine predictors.

The full set of variables within **L** and **Z** will be determined using a consensus of clinical and statistical expertise. The same will be done for the final expanded DAG depicting the causal pathways between these variables.

### Context and rationale for defining the exposure(s) of interest

The full exposure is typically several cycles of SACT over the treatment period. However, individuals may experience outcomes at any time within the treatment period, see diagram (Fig. 2). Therefore, the outcome of EUA30 may occur more than once. However, in this study we focus on the outcome of EUA30 after the first dose of the first cycle only. For the outcome of death, this will be any occurrence between the start of follow up and end of study period.

### Context and rationale for outcome(s) of interest

The outcomes of interest are:

1. Development of adverse events as captured by any unplanned or emergency admission up to 30 days after first SACT dose (EUA30).
2. Death up to 2 years after first dose of SACT

### Context and rationale for covariates

We will consider certain methods that allow the use of L variables to “deconfound” while they are not needed at deployment time, i.e. when obtainung a prediction for a new person.

Set L: confounders which can also be predictors of the outcomes.

Set Z: predictors of the outcome but not related to the exposure (treatment), i.e. not confounders.

### Data extraction

The SACT dataset collects information on the use of systemic anti-cancer therapies across all NHS England trusts. The SACT registry includes a record of all patients who received SACT along with their diagnosis and treatment regimens. Detailed information on the SACT registry may be found online via the NHS Digital collections [17]. We assume that all patients who received treatment for lung cancer would have a record in the SACT database [18].

The data were provided in the following parts:

1. Hospital Episode Statistics (HES)
  - Admitted Patient Care (HES APC)
  - Emergency department attendances (HES AE)
  - Outpatient attendances (HES OP)
  - Office for National Statistics mortality
2. National Cancer Registration and Analysis Service (NCRAS)
3. Systemic Anti Cancer Therapy (SACT)

### Sample size considerations

There are no previous studies where a counterfactual prediction model was developed for lung cancer for any outcome, which makes it difficult to calculate sample size based on previous literature. As the primary outcome is binary, we considered the feasibility of this model by assessing the variables (or parameters to be estimated) per outcome [19]. We aim for 10 variables per outcome, and where this number falls below 10 we investigate the parameters we included in the model. For the secondary outcome of survival, a similar approach will be taken.

For the primary outcome, we also used the method by Riley et al. [20] to perform a calculation of the mean absolute prediction error (MAPE) given the existing sample size. MAPE is the average error in the model’s estimated outcome probability to allow for the intended setting of the application of the model. The recommended cut off in the paper by Riley et al. was 0.05.

For a binary outcome, the MAPE can be calculated as:

$$\mathrm{In}\left(MAPE\right)=-0.508-0.544\;\mathrm{In} \left(n\right)+0.259\;\mathrm{In}\left(\varnothing \right)+0.054\; \mathrm{In}\left(\mathrm{P}\right)$$

where n is the sample size of the development dataset, $\varnothing$ is the outcome proportion $\left(\le 0.5\right)$, and $\mathrm{P}$ is the number of candidate predictor parameters$\le 30$.

Given the above equation, and the following initial known variables within the equation$n=24,000$, $\varnothing =0.34$,$P=14$, this gives a MAPE = 0.007.

### Missing data

If necessary, we will perform multiple imputations (MI) conditional on observed variables. The observed variables may be those used in the model development and other complete variables. We will combine the estimates using Rubin’s rule, or other suitable method aimed at prognostic models [21]. We will compare the estimates with those obtained from a complete records analysis if needed. We will also consider simpler methods such as missingness indicator (valid under plausible assumptions), “single imputation” using an imputation regression model, and median imputation as more practical methods which can be replicated during validation [22].

### Model development and internal validation

We will initially fit a multivariable logistic model of the outcome on the exposure and covariates. We will use the whole set for development of the prediction model and internal validation will be done using K-fold cross validation. The number of folds, K, will depend on the effective sample size, which is 5 times the number of outcomes in the dataset [23]. The validation will be on the same dataset, again using K-fold cross validation. We will assess the choice between a penalised and non-penalised logistic regression based on the magnitude of the parameter lambda.

In addition to the traditional prognostic model development, we will also apply prediction under intervention using a 4-year cohort (2016 to 2019) of linked SACT-HES lung cancer data.

For the main outcome which is the development of SAEs, the counterfactual prediction model will be using covariate set ${\boldsymbol{L}}$ and **Z** as the counfound and predictors. Some of the ${\boldsymbol{L}}$ variables will also act as predictors.

$E\left({Y}_{EUA}^{S}|L=l,Z=z\right)$ Risk of adverse events conditional on **Z** and **L**, if treatment regimen $S=A/B/C/D$ is given.

For confounding adjustment, we will use covariate set **L** to adjust for confounding in the counterfactual prediction model.

### Analysis specification

Tables 3 and 4 provide details of the proposed analysis specification and the sensitivity analyses. Immortal time between DTT and first dose of SACT was assumed to have little impact on the study conclusion for the outcome of EUA30.

**Table 3:** Primary analysis specification

Model(s): *(provide details or code)*	Multivariable logistic regression to calculate the probability of the outcome under each of the regimens A, B, C, or D. Then predicting under each hypothetical intervention A, B, C, or D Based on the best lambda value, we will make the choice to apply a penalised ridge regression to mitigate against extreme predictions in new small samples. Ridge shrinkage will drive the model coefficients towards the null thereby generating less extreme estimates of probabilities, while retaining all the covariates
Confounding adjustment method:	Potential confounding factors will be included in the prediction model
Subgroup Analyses:	None planned

**Table 4:** Sensitivity analysis

	**What is being varied? How?**	**Why?**	**Strengths of the sensitivity analysis compared to the primary**	**Limitations of the sensitivity analysis compared to the primary**
Number of deaths between DTT and first dose of SACT is > 5% of total deaths	Use clone-censor-wight methods for the outcome of death	The impact of immortal time bias on the analysis	Controls for immortal time bias	Complex and time consuming
Complete records analysis	Using complete records only to carry out the analyses for the primary and secondary outcomes	To assess if and how the conclusion varies without any imputation		

## Discussion

A diagnosis of lung cancer is an emotional burden on both the patient and their family. The prognosis of patients with advanced lung cancer is not a favourable one. The decision for best treatment choices are usually presented by the treating physician, and patients will then discuss the options with their physician in order to decide which treatment strategy they wish to opt for. Any aids to guide this decision making for both clinicians and patients would make the process more objective and less burdensome.

We developed this protocol with the aim to develop a risk score that will help patients and their clinicians make more informed treatment choices. This will be the first decision tool developed for advanced NSCLC patients using a counterfactual approach. We aim to conduct a future study to for external validation.

The benefits of this approach are that the process of decision making is incorporated in the score development, which is expected to improve external validity when the score is used in clinical practice.

A limitation of our approach is that the current assessment tools used for prediction models are not valid for counterfactual prediction. To overcome that, we are working with methodologists to apply more appropriate tools such as the counterfactual measures of calibration, discrimination, and overall prediction error for validation of predictions under interventions described by Boyer et al. [14].

## Data Availability

The data that support the findings of this study are available from [third party name] but restrictions apply to the availability of these data, which were used under license for the current study, and so are not publicly available. Data are however available from the authors upon reasonable request and with permission of [third party name].
